# The Microbial Spectrum of Neonatal Sepsis in Uganda: Recovery of Culturable Bacteria in Mother-Infant Pairs

**DOI:** 10.1371/journal.pone.0072775

**Published:** 2013-08-27

**Authors:** Julius Kiwanuka, Joel Bazira, Juliet Mwanga, Dickson Tumusiime, Eunice Nyesigire, Nkangi Lwanga, Benjamin C. Warf, Vivek Kapur, Mary Poss, Steven J. Schiff

**Affiliations:** 1 Department of Pediatrics, Mbarara University of Science and Technology, Mbarara, Uganda; 2 Department of Microbiology, Mbarara University of Science and Technology, Mbarara, Uganda; 3 Department of Neurosurgery, and Program for Global Surgery and Social Change, Department of Global Health and Social Medicine, Harvard Medical School, Children's Hospital Boston, Boston, Massachusetts, United States of America; 4 Department of Veterinary and Biomedical Sciences, Penn State University, University Park, Pennsylvania, United States of America; 5 Center for Infectious Disease Dynamics, Department Biology, Penn State University, University Park, Pennsylvania, United States of America; 6 Center for Neural Engineering, Departments of Neurosurgery, Engineering Science and Mechanics, and Physics, Penn State University, University Park, Pennsylvania, United States of America; Argonne National Laboratory, United States of America

## Abstract

Neonatal sepsis in the developing world is incompletely characterized. We seek to characterize the microbial spectrum involved in sepsis and determine the role of maternal transmission by comparing organisms that can be cultured from septic newborn infants and their mothers. From 80 consecutive mother-infant pairs meeting clinical criteria for neonatal sepsis, we collected infant blood and spinal fluid, and maternal blood and vaginal specimens. Identifiable bacteria were recovered from the blood in 32.5% of infants, and from 2.5% of cerebrospinal fluid cultures, for a total of 35% recoverable putative causative agents. Bacteria recovered from vaginal specimens were not concordant with those recovered from infants. Similarly there was no concordance of bacteria recovered from blood and cerebrospinal fluid. We conclude that relying on traditional bacterial culture techniques does not adequately delineate the role of maternal versus environmental sources of neonatal sepsis in this setting. More sensitive molecular approaches will be needed to properly characterize the maternal and environmental microbial community involved in neonatal sepsis in such developing countries.

## Background

Neonatal sepsis (NS), a severe systemic bacterial infection in newborn infants within the first 4 weeks of life, claims over 1.5 million infant lives each year, the majority in sub-Saharan Africa and southern Asia [1]. There are two classes of NS: early onset, within the first week of life presumed to be acquired from prenatal and intrapartum maternal transmission, and late onset within weeks 2–4 of life from environmental and nosocomial sources [1].

The spectrum of bacteria causing NS in industrialized countries is relatively homogenous in North America, Europe, Australia, and South Africa: group B *Streptococcus*, *Escherichia coli*, and *Listeria monocytogenes* dominate [2]. When gram-negative enteric bacteria cause NS in industrialized countries, risk factors typically include neural tube defects and urinary tract anomalies [3]. In contrast, the spectrum of bacteria causing NS in the developing world can be biased towards Gram negative organisms, and particular bacterial spectra can differ from each site where high quality (culture positive) data are available [4], [5]. There can be a striking rarity of group B *Streptococcus* (GBS) in many studies in the developing world [6], [7], and maternal carriage rates of this organism appear to differ at various sites [7], [8], [9]. Nevertheless, where GBS is the most common organism isolated in NS [10], [11], maternal carriage rates are comparable to those in industrialized countries [12]. Such conflicting findings have hampered our understanding of prevention and treatment of neonatal sepsis in the developing world.

Long-term, we seek to define the *neonatal septisome* – the sum total of the invasive microorganisms that are found within septic newborn infants. Such a broad microbial characterization goes beyond the typical assumption of single species bacterial causality of the syndrome of NS in an individual patient, and admits the possibility that NS could arise from a polymicrobial infection including both bacteria and viruses. Our broad characterization is in part motivated by the difficulties in identifying the underlying etiology of postinfectious hydrocephalus, which occurs as a sequela in a subset of survivors of NS [13].

In this report, we focus on traditional bacteriological culture techniques to assess samples from infants with NS and their mothers at the Mbarara Regional Hospital in Uganda. We correlated organisms recovered from infant blood and cerebrospinal fluid (CSF), in addition to maternal vaginal flora, in order to seek a more comprehensive characterization of the microbiological spectrum and the role of maternal transmission in NS in this setting.

## Methods

Ethics Statement: Under Institutional Review Board approval from the Mbarara University of Science and Technology, Harvard University, and Penn State University, the following study was performed. Written consent was offered in both English (the primary national language of Uganda), and Runyankore (the regional language of southwestern Uganda). Written informed consent was obtained from all participants. Further oversight for this project was provided by the Uganda National Council for Science and Technology.

The Mbarara Regional Referral Hospital is the main teaching hospital for, and is situated adjacent to, the campus of the Mbarara University of Science and Technology. It is the referral center for southwestern Uganda, and typically admits several hundred cases of presumed NS each year to its pediatric wards.

Eligibility was sought from neonates whose mothers were at least 18 years of age and who met the following inclusion criteria: 1) Infant with presumed bacterial sepsis with either 1a) fever, lethargy, and poor feeding, or 1b) hypothermia, lethargy, and poor feeding, or 1c) fever, full fontanel, and poor feeding, 2) Infant greater than 2.0 kg weight, and 3) Infant 1 month or less in age. Exclusion criteria were 1) known local infection other than sepsis, 2) known congenital malformation, 3) known cutaneous or gastrointestinal fistula, or 4) known birth trauma such as wounds or fractures. We used these relatively strict clinical criteria to maximize our yield of sick neonates who were likely to have true microbiological sepsis.

Maternal interviews were conducted in a private examination room off of the pediatrics ward dedicated to the maternal interview and specimen collection. We permitted mothers to withhold consent for having their vaginal samples collected, but permitted their infants to be studied, if that was their personal preference.

Following maternal informed consent, the following samples were collected. Lumbar punctures were performed with sterile disposable styletted neonatal spinal needles using aseptic technique, withdrawing up to 0.6 mL of CSF allocated for culture and gram stain (0.2 mL), for bacterial DNA (0.2 mL), and for viral RNA (0.2 mL). DNA and RNA specimens were collected for sequence analysis to be conducted in the future. CSF was only withdrawn as free flow from the spinal needle within 1 minute after insertion without suction, and only so long as free flow was obtained.

Blood for all required tests was collected using standard aseptic technique; withdrawing up to 1.0 mL blood, of which 0.4 mL was allocated for culture, malaria smear, and HIV testing, followed by 0.2 mL for bacterial DNA, and 0.4 mL for viral RNA.

CSF and blood samples were taken immediately (within 2 hr) upon admission, and prior to antibiotic administration. However, some neonates referred in from a community clinic/health center had received antibiotics prior to referral (31 of 80, 38.7%). No infants were directly admitted from in-hospital delivery settings, and none had an indwelling catheter prior to samples being taken.

Brain heart infusion broth contained in a universal bottle was used as the primary medium for blood cultures, incubated at 35–37°C and observed for bacterial growth after 24 hours, 48 hours, and 72 hours for up to 7 days. For those bottles that indicated evidence of bacterial growth (such as turbidity or hemolysis), Gram stain was performed and subcultures plated onto solid media that included blood agar, MacConkey agar and chocolate agar. The chocolate agar plates were also incubated under increased CO_2_ atmosphere using a candle jar to enhance growth of fastidious organisms.

CSF specimens were processed for cell counts, protein and glucose determinations, Gram stain, India ink stain, culture and sensitivity tests. Vaginal specimens were collected with sterile swabs, and Gram stain, culture and sensitivity testing performed. Culture media consisted of plates containing blood agar, MacConkey agar and chocolate agar for both CSF and vaginal specimens, and were incubated at 35–37°C for 48 hours.

Organisms were identified using morphology and staining, culture characteristics on different media plus relevant available conventional biochemical tests as follows:

### 1. Streptococci

For all suspected streptococci a Gram stain and catalase test were performed. For suspected *S. pneumoniae*, the optochin sensitivity test was performed. Facilities for the bile solubility test were not available. Only inhibition zones of 9 mm and above were considered positive. Discs for bacitracin to identify Group A *Streptococci* represented by *S. pyogenes* were not available. Streptococci were expected to be catalase negative.

### 2. Staphylococci

The coagulase test for detecting free and bound coagulase was performed to distinguish less pathogenic staphylococcus (*S. epidermidis*) from the more pathogenic staphylococcus (*S. aureus*). *S. aureus* gives a positive coagulase test. The catalase and DNAase tests were also performed. All staphylococci give a positive catalase test, and more than 50% of the coagulase positive *S. aureus* are also DNAase positive. Although coagulase negative staphylococcus can be an important pathogen in neonates [14], our blood cultures were performed on a population of infants without indwelling catheters or other foreign bodies rendering this organism an unlikely pathogen.

### 3. Gram-negative bacteria

The following tests were performed for this group of bacteria: indole, citrate utilization, urease, motility using the hanging drop method, lactose/non-lactose fermentation on MacConkey agar, oxidase test, and Kligler iron agar. The indole, urease, motility and citrate utilization tests are especially important for differentiating Klebsiella and E.coli.

Sensitivity testing was performed with Stokes' method [15].

Maternal blood was tested for HIV using the Ugandan nationally approved algorithm. The Determine® HIV 1/2/O rapid antibody test kit (Inverness Medical Japan Co.) was used as the screening test, and all positive results were confirmed using the STAT-PAK® test (Chembio Diagnostic Systems, Inc. NY, USA). Discrepant results were resolved using a tie-breaker test – Uni-Gold® HIV (Trinity Biotech plc, Bray, Ireland), and if positive, CD4 counts were performed by flow cytometry using a BD-FACSCalibur® instrument (Becton Dickinson, Franklin Lakes, NJ, USA).

## Results

Eighty consecutive mother-infant pairs (80 infants of 78 mothers – including two sets of twins) were recruited from March through November 2010. Sixty-five (81.3%) were classified as early-onset sepsis (EOS), and 19 (23.7%) late-onset sepsis (LOS), by whether their infections were manifest during or after the first week of life respectively.

Blood cultures were positive in 26/80 neonates (32.5%). These included 19 with EOS and 7 with LOS, and are summarized in Table 1. We further stratified the EOS cases into <24 hrs, 24–48 hrs, and 2–6 days, using temporal stratification within the first day classified as ‘very early onset’ sepsis [16]. The most common organism recovered was *S. aureus* (20%), followed by *E. coli* (5%), and Klebsiella sp. (2.5%); there was one group B *Streptococcus* identified (1.25%). In 3 cases, Gram negative rods were recovered which were unidentifiable (3.75%).

**Table 1:** Blood culture summary.

	Early-onset					Late-onset		Totals	
	<24 hr	24–48 hr	2–6 days	Overall n (of 19)	%	n (of 7)	%	n (of 80)	%
S. aureus	6	2	5	13	68.4	3	42.9	16	20
E. coli	1	2	0	3	15.8	1	14.3	4	5
Klebsiella sp.	1	0	0	1	5.3	1	14.3	2	2.5
unidentified coliform	1	0	0	1	5.3	2	28.6	3	3.8
Group B Streptococcus	1	0	0	1	5.3	0	0	1	1.3
**Total**	**10**	**4**	**5**	**19**		**7**		**26**	**32.5**

If we exclude the 31 neonates with antibiotic exposure we would be left with 49 infants, 19 (39%) of whom were culture positive, compared to 7 (23%) of the 31 with antibiotic exposure who were culture positive. The odds ratio is 0.46, with a 95% confidence interval of 0.17–1.28, that cultures are positive with prior antibiotic exposure. Although proportionally more infants without antibiotic exposure were culture positive, the relationship between prior exposure to antibiotics and culture positivity was not significant in this cohort.

Five infants were born to mothers known to be HIV positive (either on self-report or testing) in this cohort; 3 of these (infants) had *S. aureus* recovered from blood culture while the other 2 had negative cultures. Maternal CD4 counts were available for 2 of the 3 infants with *S. aureus* growth −442 and 705 cell/µL respectively.

The majority (67.5%) of infants meeting our clinical criteria for sepsis did not demonstrate bacteria that we could recover through blood culture. None of the blood culture-positive infants grew more than a single organism.

CSF cultures were positive in 2/80 neonates (2.5%). There was one isolate of *S. pneumoniae*, and one unidentified Gram-negative rod. These isolates were both from patients without organisms cultured from their blood.

The rate of recovery of pathogenic organisms associated with NS from blood and CSF using these methods was 35% (28/80).

Vaginal specimens were positive for potential pathogens in 25/53 mothers who consented to the collection procedure (47%, taking into account that 2 vaginal specimens were mothers of twins where both twins met our criteria for presumed sepsis). The most common organisms recovered (Table 2) were Klebsiella species (18.9%), followed by *S. aureus* (13.2%), *E. coli* (9.4%), *C. albicans* (3.8%), one isolate of a Streptococcal species (1.9%) and an unidentified Gram negative rod (1.9%).

**Table 2:** Vaginal culture summary.

	n (of 53)	%
Klebsiella sp.	10	18.9
S. aureus	7	13.2
E. coli	6	9.4
C. albicans	2	3.8
Strep sp.	1	1.9
unidentified coliform	1	1.9

The most common EOS organism recovered from the infant blood, *S. aureus*, was a frequently identified pathogen on vaginal culture, consistent with a maternal origin for some fraction of infections with this particular organism. Nevertheless, when we stratified maternal pathogens by the age of the onset of NS in cases where neonatal blood cultures were positive, no clear linkage pattern emerged (Table 3). Indeed, in 5 cases where we recovered pathogenic bacteria both from maternal vaginal specimens and infants' blood culture, none of the organisms were concordant (Table 4). In the two cases with positive CSF culture, one was from a mother without recovery of pathogens from the vaginal specimen, and one was from a mother that withheld consent for a vaginal specimen.

**Table 3:** Stratification vaginal culture results for all infants with positive blood cultures.

	Early-onset					Late-onset		Totals	
	<24 hr	24–48 hr	2–6 days	n	(% of 19)	n	(% of 7)	n	(% of 80)
S. aureus	0	0	0	0	0	1	14.3	1	1.25
E. coli	2	0	0	2	10.5	1	14.3	3	3.75
Klebsiella sp.	1	0	0	1	5.3	0	0	1	1.25
unidentified coliform	0	0	0	0	0	0	0	0	0
Streptococcus spp.	0	0	1	1	5.3	0	0	1	1.25
No bacterial growth	3	3	2	8	42.1	5	71.4	13	16.25
No consent for swab	3	1	2	6	31.6	1	14.3	7	8.75
Not done	1	0	0	1	5.3	0	0	1	1.25
**Total**	**10**	**4**	**5**	**19**		**7**		**26** *	**32.5** *

* One mother had a mixed growth of E. coli and *Staphylococcus aureus*, and the totals are adjusted for this.

**Table 4:** Comparison between maternal and infants' organisms.

	Patient ID	Mother's vaginal swab culture	Infant's blood culture	Infant's CSF culture
1	NS008	E. coli	S. aureus	No growth
2	NS012	E. coli	S. aureus	No growth
3	NS019	Mixed growth - E. coli/S. aureus	Unidentified coliform	No growth
4	NS026	Klebsiella	S. aureus	No growth
5	NS035	Streptococcus sp.	S. aureus	No growth

Table represents patients in whom a potential pathogen was recovered from both the infant and the mother.

## Discussion

Neonatal sepsis is common in developing countries, but in the studies of which we are aware, the putative causative agents are often not identified from blood using bacterial culture. Our recovery rate of pathogens in 32.5% of such infants is among the higher percentages reported, and very similar to a comparable study performed at the Mulago Hospital in Kampala where 37% of infants had positive blood cultures [17], and to reports from Nigeria [18] and India [19]. Our combined rate of organism recovery from blood and CSF was 35%.

These rates of organism recovery were similar despite very different blood volumes allocated for blood culture in the two Ugandan series (0.4 vs 2.0 ml in ours vs the Mulago patients). We constrained our volumes of blood obtained for culture by both safety considerations in the structure of our proposal (1 ml total blood was permitted), and our intention to broaden our testing of blood specimens beyond bacterial culture (0.4 ml were allocated for culture, malaria smear, and HIV testing). It is well established that if the number of colony forming units per ml of blood approaches small numbers, that the reliability of blood culture decreases [20], [21], [22]. The tradeoff in the developing world is that if the variety and breadth of the spectrum of fastidious or difficult to culture organisms is increased, then molecular strategies [23] may increase the identification rate. Below a certain number of colony forming units per ml, small volume analysis will fail for all techniques. But the similar rates of recovery, and the similar spectrum of organisms in these two Ugandan series, suggests that volume plays a considerably smaller role than the spectrum of the organisms involved. Although there must be a small effect from the difference in sample volumes, it does not appear to be substantial in that both series fail to recover culturable organisms in a comparable majority of cases of clinical NS.

Another factor reducing the rate of organism recovery is the inconsistent and largely undocumented administration of antibiotics to 38.7% of these neonates prior to arrival at the Mbarara Regional Hospital. Of this cohort, failure of organism recovery through culture was 77% (24 of 31). Such partial treatment of active infection should disproportionately reduce the organism recovery rate by culture, with less effect on identification through molecular approaches. However, in our cohort, the rate of recovery of culturable organisms was not significantly different in the infants who received antibiotics.

We designed our inclusion criteria to maximize the inclusion of neonates with true microbial sepsis, but we recognize that some of these infants will suffer from noninfectious syndromes such as hypoxic-ischemic encephalopathy. We anticipate that the fraction of false positive diagnoses of microbial sepsis is low in our sampled population, but we remain unable to quantitatively estimate the true fraction of uninfected neonates in this cohort.

Our blood culture findings were dominated by *S. aureus*, *E. Coli*, and Klebsiella species, organisms that have been described in different combinations as major causes of NS in developing countries [1]. Our particular combination is very similar to the spectrum of organisms recovered from neonatal blood in the Mulago Hospital series in Uganda [17]. With the exception of the occasional report (such as the large series from Malawi [8], [11]), in many settings of infants with NS in developing countries the recovery of group B Streptococci appears unusual [17]. It remains unclear why rates of group B streptococcal sepsis are more uniformly prominent in sepsis in the first week of life in industrial countries [24]. In parallel with sepsis rates, the maternal carriage rates of group B *Streptococci* can differ in developing and industrialized countries and can vary at different sites [11]. In our series, we recovered only 1 case of group B *Streptococcus* on blood culture, and recovered Streptococcal species from only one vaginal culture. Our findings are consistent with a low rate of group B *Streptococcal* NS correlating with a low rate of maternal vaginal carriage in this southwestern Ugandan population.

It is of concern that births in hospitals in the developing world may be settings for acquisition of *S. aureus* and *K. pneumoniae* infections from hygiene practices and common source infections [25]. Although in principle we may be able in the future to validate maternal transmission from genetic fingerprinting of bacteria found in both mother and infant, we are presently unable to distinguish between maternal and caregiver sources. In addition, the vast majority of births in Uganda are out of hospital, where hygiene practices will carry infection risks quite distinct from those documented within hospital settings [25]. Our findings of discordant culture results in early sepsis between infants and maternal cultures are consistent with a contribution from non-maternal peripartum hygiene effects.

To our knowledge, this is the first study of NS in a developing country where an attempt has been made to correlate infant blood and CSF organisms with maternal vaginal flora to ascertain the role of maternal transmission at birth to NS. Although a broadly similar range of organisms was recovered from infants' blood and maternal vaginal specimens, we found no concordance between organisms recovered from blood and vaginal cultures in the same mother-infant pairs. Culture data is biased towards organisms that grow most readily in the available culture media, and this undoubtedly contributes to the similar spectrum of organisms recovered.

The EOS patients had a higher predominance of *S. aureus* than LOS patients (68.4 vs 42.9%). *S. aureus* was one of the more common pathogens recovered from maternal vaginal sampling (13%), and although not concordant, neither are our findings inconsistent with a maternal source of this agent for EOS cases. It is also possible that maternal flora changes substantially in the days following birth, progressively obscuring the source of causative pathogens with delayed vaginal bacterial culture. Nevertheless, we anticipate, especially in the EOS cases (>80% of our cases), that it should be possible to recover genomic sequences remaining from pathogens that may have been transmitted to the infant during birth. The time-scale beyond which such evidence of maternal carriage becomes undetectable, with culture and molecular techniques, is presently unknown.

In the present study, we found only two cases with evidence of meningitis, and both patients had negative blood cultures. The treatment of NS involving meningitis should be significantly more prolonged than for NS that does not involve the nervous system [2]. Nevertheless, performing lumbar puncture in developing world settings adds to the complexity and expense of evaluating infants with NS. Our finding of negative blood cultures in the presence of culture-positive neonatal meningitis further emphasizes the potential unreliability of blood culture as a screening tool for infection in the CSF in NS, and the potential value of routine lumbar puncture in such settings.

In related work, we have identified a population of infants surviving NS in Uganda who develop postinfectious hydrocephalus (PIH) [26]. These infants often demonstrate a significant degree of brain destruction as a consequence of prior NS, and typically give a history of convulsions during the previous septic episode [26]. Although we have been unsuccessful in recovering organisms from the CSF of such infants with PIH using bacterial culture, sequencing the 16S ribosomal DNA from the CSF reveals a predominance of sequences from Gram-negative bacteria, especially *Acinetobacter*, *Klebsiella*, and *E. coli* species [13]. Consistent with the present study based on bacterial culture, it was uncommon to detect DNA fragments from streptococcal species in PIH infants in Uganda [13]. A recently confirmed link between rainfall climate dynamics and NS cases leading to PIH [27], supports an environmental source for an important fraction of PIH cases. A long-term survival analysis [28] and an economic burden of disease analysis [29] demonstrate the severe consequences for this cohort of survivors of NS. Nevertheless, this present NS study does not further identify the agents responsible for these post-sepsis hydrocephalic sequelae.

Neonatal sepsis is a serious disorder where there is substantial morbidity in the survivors. The optimal approach to these infants is prevention. Prevention requires that we identify the routes of infection, and either institute appropriate antimicrobial prophylaxis if maternally transmitted at birth, or create public health interventions if infections occur in the infants' home environments. Properly assessing the causes of NS will require that we identify the putative causal agents responsible for this disorder.

## Conclusions

Our findings, and the literature of NS from the developing world, suggest that traditional bacterial culture techniques may be failing to identify a substantial fraction of the causal agents of neonatal sepsis. Furthermore, bacterial culture appears unable to suitably delineate the roles of maternal versus environmental sources of infection in this setting. Alternative molecular technologies may therefore have a valuable role to play in characterizing the microbial spectra associated with neonatal sepsis [30], which will be a prerequisite for better treatment and prevention of these infections.
